# Multi-vessel coronary artery grafting: analyzing the minimally invasive approach and its safety

**DOI:** 10.3389/fcvm.2024.1391881

**Published:** 2024-05-07

**Authors:** Ryohei Ushioda, Aina Hirofuji, Dit Yoongtong, Boonsap Sakboon, Jaroen Cheewinmethasiri, Thanin Lokeskrawee, Jayanton Patumanond, Suppachai Lawanaskol, Hiroyuki Kamiya, Nuttapon Arayawudhikul

**Affiliations:** ^1^ Cardiovascular and Thoracic Surgery Unit, Department of Surgery, Lampang Hospital, Lampang, Thailand; ^2^ Department of Cardiac Surgery, Asahikawa Medical University, Asahikawa, Japan; ^3^ Department of Emergency Medicine, Lampang Hospital, Lampang, Thailand; ^4^ Center for Clinical Epidemiology and Clinical Statistics, Faculty of Medicine, Chiang Mai University, Chiang Mai, Thailand; ^5^ Chaiprakarn Hospital, Chiang Mai, Thailand

**Keywords:** intercostal space, left small thoracotomy, median sternotomy, minimally invasive direct coronary artery bypass grafting, surgical revascularization

## Abstract

**Introduction:**

At our institution, we perform off-pump coronary artery bypass (OPCAB) as a standard procedure. Moreover, patients with favorable coronary anatomy and condition are selected for minimally invasive cardiac surgery (MICS)-OPCAB. We retrospectively compared early outcomes, focusing on safety, between MICS-OPCAB and conventional off-pump techniques for multivessel coronary artery bypass grafting (CABG).

**Methods:**

From August 2017 to September 2022, 1,220 patients underwent multivessel coronary artery grafting at our institution. They were divided into the MICS-OPCAB group (MICS group = 163 patients) and the conventional OPCAB group (MS group = 1057 patients). Propensity score matching (1 : 1 ratio) was applied to the MICS-OPCAB and MS groups (149 patients per group) based on 23 preoperative clinical characteristics.

**Results:**

After matching, there were no significant differences in preoperative characteristics between the groups. The MICS group had a lower total graft number (2.3 ± 0.6 vs. 2.9 ± 0.8, *p* < 0.001) and fewer distal anastomoses (2.7 ± 0.8 vs. 3.2 ± 0.9, *p* < 0.001). There were no significant differences in hospital stay, intensive care unit stay, postoperative complications, and 30-day mortality. The MICS group had less drain output (MICS 350 ml [250–500], MS 450 ml [300–550]; *p* = 0.013). Kaplan–Meier analysis revealed no significant differences in postoperative MACCE (major adverse cardiac or cerebrovascular events)-free and survival rates between the groups (MACCE-free rate *p* = 0.945, survival rate *p* = 0.374).

**Conclusion:**

With proper patient selection, MICS-OPCAB can provide good short to mid-term results, similar to those of conventional OPCAB.

## 1 Introduction

Minimally invasive direct coronary artery bypass grafting (MIDCAB) (1) was popularized in the 1990s. Nowadays, multivessel coronary artery bypass grafting (CABG) via a left small thoracotomy has developed as a minimally invasive cardiac surgery coronary artery bypass (MICS-CABG) and maintained gaining attention (2, 3). In our institution, we have performed off-pump coronary artery bypass (OPCAB) as a standard procedure, and patients with favorable coronary anatomy and condition are selected for MICS-OPCAB. This study retrospectively compared the early outcomes of MICS-OPCAB and conventional off-pump techniques regarding the safety of treating multivessel coronary disease.

## 2 Patients and methods

**Figure 1:**
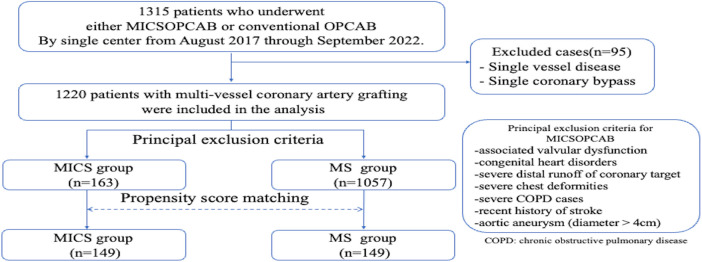
Study protocol.

We retrospectively analyzed 1,315 patients who underwent CABG between August 2017 and September 2022 at our single center (Figure 1). After excluding patients with single-vessel disease, or single coronary bypass (*n* = 95), a total of 1,220 patients who underwent multivessel CABG were included in this analysis. Patients were divided into the MICS-OPCAB (MICS group = 163 patients) and conventional OPCAB (MS group = 1,057 patients) groups. The MICS-OPCAB group was propensity score-matched (PSM) with the OPCAB group in a 1 : 1 ratio (MICS = 149, MS = 149), where matching was performed based on 23 covariates of preoperative clinical characteristics.

Within one week post operation, coronary computed tomography angiography (CTA) was performed on all MICS-OPCAB patients with creatinine levels of <1.5 ng/dl; while in the MS group, CTA was only considered for complex bypass cases or discharged patients who developed clinical symptoms indicative of postoperative cardiac ischemia.

### 2.1 Inclusion and exclusion criteria

Our institution has five surgeons total, where one senior surgeon performed all MICS-OPCAB, and the other surgeons performed conventional OPCAB. The principal exclusion criteria for MICS-OPCAB were: comorbid valvular dysfunction, congenital heart disorders, severe distal runoff of coronary target, severe chest deformities, severe chronic obstructive pulmonary disease (COPD), stroke within four weeks prior to surgery, and aortic aneurysm (diameter >4 cm). Relative contraindications included current smokers, tuberculosis, interstitial lung disease history, morbid obesity, and previous cardiac surgery. Acute myocardial infarction within seven days, poor left ventricular ejection fraction (LVEF) < 20%, or cardiomegaly (left ventricular end-diastolic diameter >6.0 cm) were only precautionary, and included in our study.

### 2.2 Surgical technique

Conventional OPCAB was performed via median sternotomy. Technical details of our MICS-OPCAB techniques were described in our previous report (4). Shortly, patients were positioned with the left chest elevated 30 to 40 degrees to widen intercostal space (ICS). All procedures were performed under differential ventilation, through an 8–10 cm left mini-thoracotomy (with 1/3 of the incision medial to the mid-clavicular line) in the fourth or fifth ICS to expose both internal thoracic arteries. The left internal thoracic artery (LITA) and right internal thoracic artery were harvested full length in a skeletonized fashion under direct vision. The saphenous vein graft was harvested non-touch or skeletonized by skip incision. Usual sequence of anastomosis was the left anterior descending artery (LAD), obtuse marginal branch, and the last posterior descending artery or posterior-lateral artery. Two deep pericardial stitches were routinely fixed in order to expose the lateral and inferior walls of the heart. On-pump conversion was considered in cases with unstable hemodynamics or multiple ventricular arrhythmias.

### 2.3 Follow-up

Patients were followed up every six months at our outpatient clinic. Information on all causes of death and cardiac complications during the follow-up period was obtained from Lampang Hospital's database. We achieved a 100% follow-up rate by contacting both the patients and their families for any missing data.

### 2.4 Statistical analysis

Group assignments were not random because the operative approach was a matter of subjective choice. Therefore, we calculated standardized mean differences before and after PSM to assess the balance of variables between the groups. The propensity score (PS) was obtained from a logistic regression model, including 23 covariables presented in Table 1 without European System for Cardiac Operative Risk Evaluation (Euro SCORE) Ⅱ, Society of Thoracic Surgeons (STS) score, and Emergent case (Table 2). Patients were matched 1:1 using the nearest neighbor matching method without replacement and a caliper width of 0.2 of the standard deviation of the logit of the estimated PS. A double adjustment was performed in case of remaining imbalance that standardized differences over 0.10. For double adjustment in the matched sample, we utilized Cox PH regression. Continuous variables exhibiting a normal distribution were tested using the *t*-test, and continuous variables exhibiting a non-normal distribution were tested using the Mann–Whitney *U*-test. For categorical variables, chi-squared or Fisher's test was used. In the final analysis, the categorical endpoint was tested using McNemar's exact test to incorporate the correlation after matching. Statistical significance was set at *p* < 0.05. The Kaplan–Meier method was used to demonstrate survival rate and freedom from major adverse cardiac or cerebrovascular events (MACCE). In addition, Cox PH regression analysis was used to evaluate the treatment effect to free MACCE and survival rate, which was presented as the hazard ratio (HR) with 95% CI. The STATA Software/MP, Version 17.0 (Stata Corporation, College Station, Texas, USA) was used for the statistical analyses.

**Table 1:** Patients’ characteristics and preoperative data.

	Before PSM			After PSM		
	MICS group (*n* = 163)	MS group (*n* = 1,057)	SMD	MICS group (*n* = 149)	MS group (*n* = 149)	SMD
Age, mean ± SD years	65.2 ± 7.9	65.3 ± 8.3	0.016	65.1 ± 8.1	65.9 ± 8.5	0.091
Male gender, *n* (%)	118 (72.4)	632 (59.8)	0.269	105 (70.5)	96 (64.4)	−0.129
BMI, mean ± SD kg/m^2^	22.7 ± 3.6	23.2 ± 3.9	0.133	22.8 ± 3.7	22.3 ± 3.1	−0.139
NYHA class ≧Ⅲ, *n* (%)	24 (14.7)	400 (37.8)	0.544	24 (16.1)	18 (12.1)	−0.116
STS SCORE, median [IQR]	1.56 [0.90–2.50]	1.82 [1.11–3.28]	0.229	1.57 [1.00–2.50]	1.58 [1.03–2.59]	−0.014
Euro SCORE, median [IQR]	1.39 [0.94–2.36]	1.87 [1.10–3.80]	0.299	1.40 [0.97–2.63]	1.68 [1.06–2.70]	0.086
Comorbidity, *n* (%)						
Hyperlipidemia	160 (98.2)	1,037 (98.1)	0.004	146 (98.0)	145 (97.3)	−0.044
Hypertension	161 (98.8)	1,042 (98.6)	0.017	147 (98.7)	147 (98.7)	<0.001
Diabetes mellitus	80 (49.1)	534 (5.5)	0.029	74 (49.6)	72 (48.3)	−0.027
Chronic renal disease (Cr ≧ 1.5 ng/dl)	22 (13.5)	213 (20.2)	0.179	21 (14.1)	25 (16.8)	0.074
Dialysis	18 (11.0)	124 (11.7)	0.022	17 (11.4)	24 (16.1)	0.136
COPD	9 (5.5)	113 (10.7)	0.190	8 (5.4)	11 (7.4)	0.082
Cerebral vascular accident	11 (6.7)	74 (7.0)	0.010	11 (7.4)	10 (6.7)	−0.026
PAD	11 (6.7)	139 (13.2)	0.215	11 (7.4)	12 (8.1)	0.025
STEMI	23 (14.1)	167 (15.8)	0.050	23 (15.4)	19 (12.8)	−0.077
Recent myocardial infarction	90 (55.2)	560 (53.0)	0.045	81 (54.4)	77 (51.7)	−0.054
Double vessel disease	70 (42.9)	97 (9.2)	0.833	56 (37.6)	51 (34.2)	−0.070
Triple vessel disease	93 (57.1)	951 (90.0)	0.804	93 (62.4)	97 (65.1)	0.056
Left main trunk lesions	74 (45.4)	407 (38.5)	0.140	61 (43.9)	61 (43.9)	−0.014
Preoperation PCI	13 (8.0)	94 (8.9)	0.033	12 (8.1)	10 (6.7)	−0.051
Preoperation IABP	7 (4.3)	208 (19.7)	0.490	7 (4.7)	7 (4.7)	<0.001
Echocardiography						
LVEF, mean ± SD %	55.4 ± 12.7	49.4 ± 16.5	0.403	54.6 ± 12.8	56.1 ± 14.6	0.109
Urgency, *n* (%)						
Elective	140 (85.9)	790 (74.7)	0.283	129 (84.6)	129 (86.6)	0.057
Urgent	23 (14.1)	255 (24.1)	0.257	22 (14.8)	20 (13.4)	−0.038
Emergent	0 (0)	6 (0.6)	0.107	0 (0)	0 (0)	
Salvage	2 (1.2)	11 (1.0)	0.018	2 (1.3)	2 (1.3)	>0.99

BMI, body mass index; NYHA, New York Heart Association; STS, society of thoracic surgeons; Euro SCORE, European system for cardiac operative risk evaluation; COPD, chronic obstructive pulmonary disease; PAD, peripheral arterial disease; STEMI, ST-elevation myocardial infarction; PCI, percutaneous coronary intervention; IABP, intra-aortic balloon pumping; LVEF, left ventricular ejection fraction.

**Table 2:** Derivation of propensity score equation from pre-treatment covariates under multivariable binary logistic regression.

Pre-treatment covariates	Coefficient	95% confidence interval	*p*-value
Age, year	−0.01	−0.03, 0.02	0.673
Male gender	0.87	0.45, 1.29	<0.001
BMI, kg/m^2^	−0.08	−0.13, −0.02	0.006
NYHA class (≧Ⅲ), *n* (%)	−1.03	−1.62, −0.45	<0.001
Comorbidity, *n* (%)			
Hyperlipidemia	−0.43	−1.89, 1.03	0.565
Hypertension	−0.31	−2.04, 1.42	0.726
Diabetes mellitus	0.21	−0.17, 0.59	0.282
Chronic renal disease (Cr ≧ 1.5)	−0.72	−1.61, 0.17	0.114
Dialysis	0.98	−0.03, 1.98	0.056
COPD	−0.49	−1.27, 0.29	0.219
Cerebral vascular accident	0.15	−0.61, 0.90	0.703
PAD	−0.38	−1.13, 0.37	0.317
STEMI	−0.23	−0.78, 0.32	0.413
Recent myocardial infarction	0.37	−0.03, 0.77	0.071
Double vessel disease	17.82	14.46, 21.18	<0.001
Triple vessel disease	15.78	12.43, 19.12	<0.001
Left main trunk lesions	0.38	<0.01, 0.76	0.049
Preoperation PCI	<0.01	−0.69, 0.70	0.992
Preoperation IABP	−1.11	−2.05, −0.18	0.020
Echocardiography			
Ejection fraction, ±SD %	1.95	0.62, 3.28	0.004
Urgency, *n* (%)			
Elective	1.56	−0.43, 3.54	0.124
Urgent	1.45	−0.49, 3.39	0.143
Salvage	3.66	1.14, 6.18	0.004

BMI, body mass index; NYHA, New York Heart Association; COPD, chronic obstructive pulmonary disease; PAD, peripheral arterial disease; STEMI, ST-elevation myocardial infarction; PCI, percutaneous coronary intervention; IABP, intra-aortic balloon pumping; LVEF, left ventricular ejection fraction.

## 3 Results

### 3.1 Preoperative characteristics

Preoperative characteristics are summarized in Table 1. Before PSM, preoperative conditions were worse in the MS group than the MICS group; the STS SCORE (*p* = 0.005), Euro SCORE II (*p* < 0.001), and number of urgent cases (*p* = 0.005) were higher in the MS group. There were no significant differences in data after PSM. However, for variables (male, body mass index, NYHA class over 3, dialysis, and LVEF) with standardized mean difference values over 10% post matching, a double adjustment was performed above five items to generate Tables 3, 4.

**Table 3:** Operative data of paired groups.

	MICS group (*n* = 149)	MS group (*n* = 149)	*p*-value
Operating time, min	247.9 ± 71.8	246.2 ± 64.2	0.828
Total grafts, average	2.3 ± 0.6	2.9 ± 0.8	<0.001
Number of distal anastomoses, average	2.7 ± 0.8	3.2 ± 0.9	<0.001
Total arterial revascularization, *n* (%)	48 (32.2)	53 (35.5)	0.625
Endarterectomy, *n* (%)	0 (0)	3 (2.0)	0.247
Perioperative transfusion, *n* (%)	84 (56.4)	100 (67.1)	0.074
Complete revascularization, *n* (%)	138 (92.6)	147 (98.7)	0.521
The index of revascularisation, [IQR]	1 [1–1]	1 [1–1]	0.011
Conversion to CPB, *n* (%)	0 (0)	1 (0.7)	1.000
Conversion to sternotomy, *n* (%)	2 (1.3)		
Graft, *n* (%)			
LITA	149 (100)	145 (97.3)	0.122
RITA	18 (12.1)	60 (40.3)	<0.001
BITA	18 (12.1)	59 (39.6)	<0.001
Radial artery	42 (28.2)	62 (41.6)	0.021
Gastroepiploic artery	6 (4.0)	14 (9.4)	0.103
Saphenous vein	104 (69.8)	94(63.1)	0.270

CPB, cardiopulmonary bypass; LITA, left internal thoracic artery; RITA, right internal thoracic artery; BITA, bilateral internal thoracic artery.

**Table 4:** Postoperative short-outcomes of paired groups.

	MICS group (*n* = 149)	MS group (*n* = 149)	*p*-value
Median ICU stay, days [IQR]	2 [1–2]	2 [1–3]	0.137
Hospital stay, days	6.3 ± 2.4	5.9 ± 2.5	0.148
Early extubation (≦24 h), *n* (%)	135 (90.6)	138 (92.6)	0.677
Median drain contents, ml [IQR]	350 [250–500]	450 [300–550]	0.013
30 days mortality, *n* (%)	2 (1.3)	4 (2.7)	0.684
Postoperative complications, *n* (%)			
New stroke	1 (0.7)	2 (1.3)	1.000
New dialysis	0 (0)	0 (0)	
New onset atrial fibrillation/flutter	29 (19.5)	33 (22.2)	0.669
Infection of wound	0 (0)	2 (1.3)	0.498
Reoperation of bleeding	6 (4.0)	1 (0.7)	0.121
Patients with in-hospital CTA, *n* (%)	104 (69.8)	9 (6.0)	<0.001
Total graft in-hospital patency, *n* (%)	94 (90.4)	6 (66.8)	0.067
LIMA-LAD in-hospital patency, *n* (%)	93 (96.9)	5 (100)	1.000
Patients with follow-up CTA, *n* (%)	136 (91.3)	45 (30.2)	<0.001
Median coronary follow-up days [IQR]	5 [5–14]	495 [114–838]	<0.001
Total graft patency, n(%)	118 (86.7)	33 (73.3)	
LIMA-LAD patency, *n* (%)	123 (96.1)	30 (96.8)	
Median follow-up days, [IQR]	472 [160–830]	676 [237–1,307]	0.002
MACCE long-term, *n* (%)	13 (8.7)	17 (11.4)	0.564
Cardiac death, n(%)	5 (3.4)	9 (6.0)	0.413
Peri-operation MI, *n* (%)	1(0.7)	2(1.3)	1.000

ICU, intensive care unit; CTA, computed tomography angiography; MACCE, major adverse cardiac or cerebrovascular events; MI, myocardial infarction.

### 3.2 Operative data

Operative data of the groups are shown in Table 3. There were no differences in operative time, number of complete revascularizations, nor perioperative transfusion rate, but the total graft number (MICS group 2.3 ± 0.6, MS 2.9 ± 0.8; *p* < 0.001), mean number of distal anastomoses (MICS 2.7 ± 0.8, MS 3.2 ± 0.9; *p* < 0.001), bilateral internal thoracic artery (BITA) usage rate (MICS 11.5%, MS 36.7%; *p* < 0.001) were higher in the MS group.

### 3.3 Short-term outcomes

Short-term outcomes are shown in Table 4. There were no differences in hospital stay, intensive care unit (ICU) stay, postoperative complications, nor 30-day mortality, but the MICS group had less drainage (MICS 350 (250–500) ml, MS 450 (300–550) ml; *p* = 0.013).

### 3.4 Postoperative freedom from MACCE and survival rates

**Figure 2:**
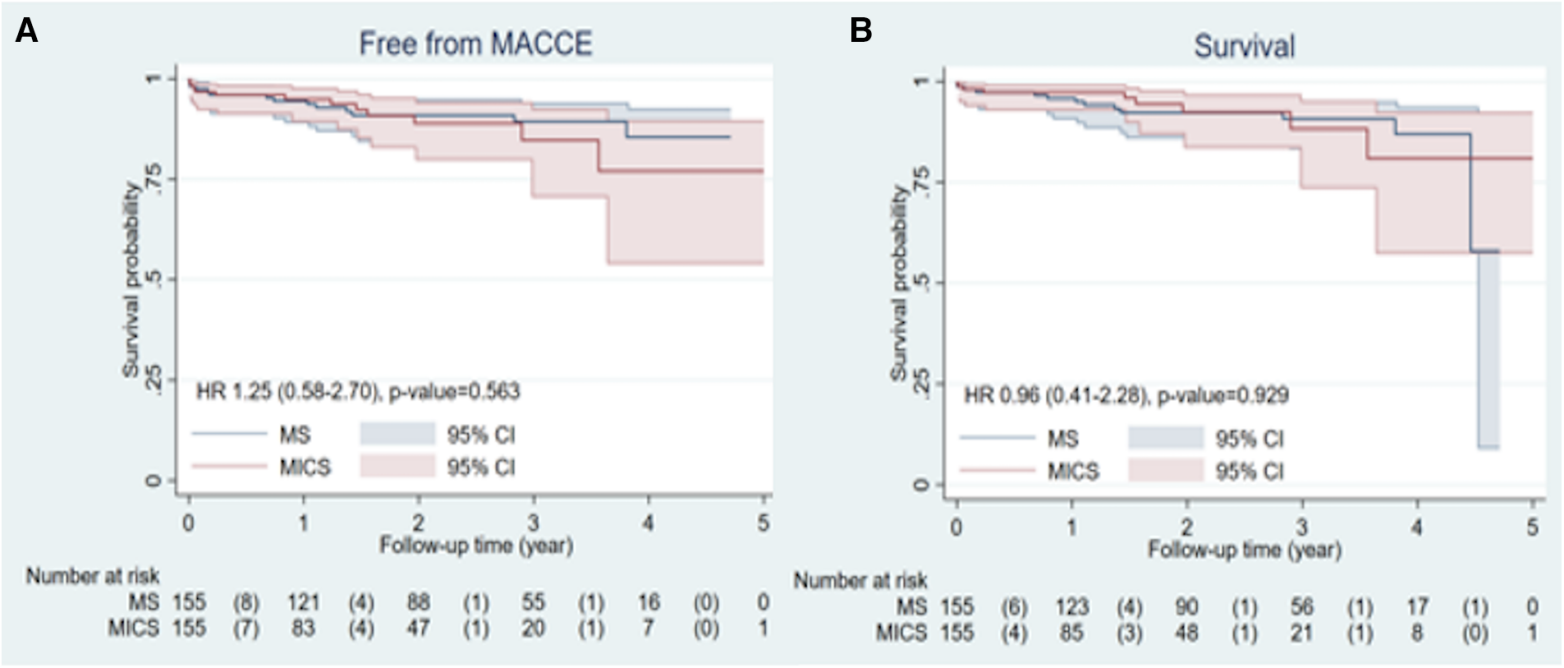
(**A**) MACCE-free (*p* = 0.945) rates, and (**B**) survival rates after operations (*p* = 0.374).

The Kaplan–Meier curve of the postoperative MACCE-free rates and survival rates are shown in Figure 2, where there were no differences between the two groups (MACCE-free rate: HR = 1.25, 95% CI: 0.58–2.70, *p* = 0.945; Survival rate: HR = 0.96, 95% CI: 0.41–2.28, *p* = 0.374).

Additionally, there were no differences in the MACCE-free rate and survival rate at 1 and 3 years as shown in Table 5.

**Table 5:** MACCE-free rates and survival rates at 1, and 3 years.

	MICS group (*n* = 149)	MS group (*n* = 149)	Hazard rate	95% confidence interval	*p*-value
1-year MACCE free rate, *n* (%)	142 (95.3)	140 (94.0)	0.78	0.29, 2.10	0.628
3-year MACCE free rate, *n* (%)	137 (92.0)	136 (91.3)	1.07	0.49, 2.36	0.868
1-year survival rate, *n* (%)	145 (97.3)	142 (95.3)	0.58	0.17, 1.97	0.375
3-year survival rate, *n* (%)	141 (94.6)	138 (92.6)	0.87	0.35, 2.18	0.770

MACCE, major adverse cardiac or cerebrovascular events.

## 4 Discussion

These results demonstrate that MICS-OPCAB is a safe and practical procedure with acceptable short and mid-term outcomes, similar to those of conventional OPCAB. The only statistically significant advantage of MICS-OPCAB was the amount of postoperative drainage.

### 4.1 Is MICS-OPCAB less invasive compared to conventional OPCAB with median sternotomy?

Previous studies report that MICS-OPCAB could lead to early extubation, less wound infections, less transfusion, faster postoperative recovery, and shorter ICU stay and hospitalization, while maintaining safety and effectiveness compared to median sternotomy (5–7). Another study (8) comparing clinical outcomes of MICS-CABG and OPCAB found that MICS-CABG led to a significantly shorter hospital stay (MICS-CABG = 4 days vs. OPCAB = 5 days) and earlier extubation (MICS-CABG = 70.0% vs. OPCAB = 12.7%) than OPCAB. However, the present study showed multivessel MICS-OPCAB to have a significant advantage only in postoperative drainage; MICS-OPCAB did not improve duration of ICU and hospital stay, perioperative transfusion amount, nor complication rates. Our perioperative transfusion rate is higher compared to rates reported for OPCAB (MICS-CABG = 56.4% vs. OPCAB = 67.1%), likely because we did not specifically focus on minimizing transfusions. Additionally, the introduction of MICS-OPCAB at our hospital in 2017 may have influenced the transfusion rates, as there could have been a higher utilization of transfusions in the MICS-CABG group to prevent conversions to median sternotomy or on-pump procedures. While MICS OPCAB may indeed offer potential improvements in post-operative quality of life for patients, as highlighted by ongoing research such as the Minimally Invasive coronary surgery compared to STernotomy coronary artery bypass grafting (MIST) trial (9), it's important to note that factors such as time to ambulation and pain assessment were not investigated in our study. Furthermore, the durations of early extubation, ICU stay, and hospitalization in the median sternotomy group were already sufficiently short in our study, which may have contributed to the lack of significant differences observed between groups. Therefore, MICS-OPCAB may truly be a less invasive procedure that does not compromise patients’ clinical outcomes.

### 4.2 Concern about the number of distal anastomoses and complete revascularization rate in MICS-OPCAB

Generally, the number of grafts and distal anastomoses in MICS-OPCAB is fewer, while operative time is longer than conventional OPCAB. Rogers et al. compared OPCAB by left anterolateral thoracotomy (ThoraCAB) and OPCAB by conventional median sternotomy (MS group) at two centers in an RCT trial, where they found the operative time was longer in the ThoraCAB group (4.1 h) than in the MS group (3.3 h) (10). Also, less patients in the ThoraCAB group had more than three grafts (2%), compared to the MS group (17%). This could be attributed to limited range of anastomosis within a constrained space, which isa disadvantage of MICS-OPCAB. Similarly, in our study, the total graft and mean number of distal anastomoses were significantly higher in the MS group, without significant differences in operative time between the two groups. While the numbers of anastomoses and grafts are generally smaller, complete revascularization can be achieved even with the MICS approach. Oleksandr et al. achieved complete revascularization by MICS-CABG on non-selected consecutive patients, using a Chitwood clamp and blood cardioplegia (11). They concluded that complete revascularization in MICS-CABG is possible without patient selection based on graft number, coronary artery quality, location, left ventricular function, age, sex, nor body mass index.

At our institution, we have consistently prioritized comprehensive revascularization with MICS-OPCAB, and our analysis showed that the complete revascularization rate was similar to that of conventional OPCAB (MICS group = 92.6% vs. MS group = 98.7%, *p* = 0.521). Although Oleksandr et al. found patient selection for MICS-CABG to be more arbitrary, we think that the critical factor for a successful MICS-OPCAB lies in the careful selection of patients for complete revascularization with minimal anastomoses and grafts.

### 4.3 Long-term outcome of MICS-OPCAB

Although it is widely accepted that MICS-OPCAB yields similar short to mid-term results to MS, the long-term outcome remains uncertain. Barsoum and colleagues reported that patients aged ≥75 years have a significantly lower 5-year all-cause mortality with MICS-CABG than with MS (12). Florisson and colleagues concluded from 12 reports from 1999 to 2017 that MIDCAB is associated with greater morbidity and reintervention rates than OPCAB by MS, due to MIDCAB having a lower rate of complete revascularization (13). However, these studies were conducted approximately 10 years ago, and with technical improvements in recent years, the clinical outcomes have also improved. In our study, there were no significant differences in the MACCE-free and survival rates after 1 year and 3 years between MICS-OPCAB and conventional OPCAB. We have interpreted these results as follows: MICS-OPCAB can provide similar long-term outcomes to conventional OPCAB if complete revascularization can be achieved.

### 4.4 Grafting strategies in MICS-OPCAB

The concept of surgical revascularization through a left thoracic minimal incision stepped up from single bypasses to multiple bypasses with total arterial grafting using BITA (3, 14, 15). Kikuchi and colleagues analyzed short-term outcomes of MICS-OPCAB using BITA and a single internal thoracic artery (SITA) at a single Japanese medical center between February 2012 and December 2015 (15). Although mean operation time was longer in the BITA group (SITA 265 ± 104 min, BITA 336 ± 73 min), all BITA grafts were harvested without major complications and patent on one-week postoperative CTAs, concluding BITA harvest to be safe in MICS-OPCAB. Based on such findings and improved long-term results with BITA (16), we have been proactively using BITA in MICS-OPCAB for young patients. However, the MICS BITA rate was lower than that of the OPCAB group in our study, because harvesting the right internal thoracic artery (RITA) in the MICS group was often difficult due to cardiac enlargement or concaved thorax (MICS 12.1%, MS 39.6%; *p* < 0.001).

**Figure 3:**
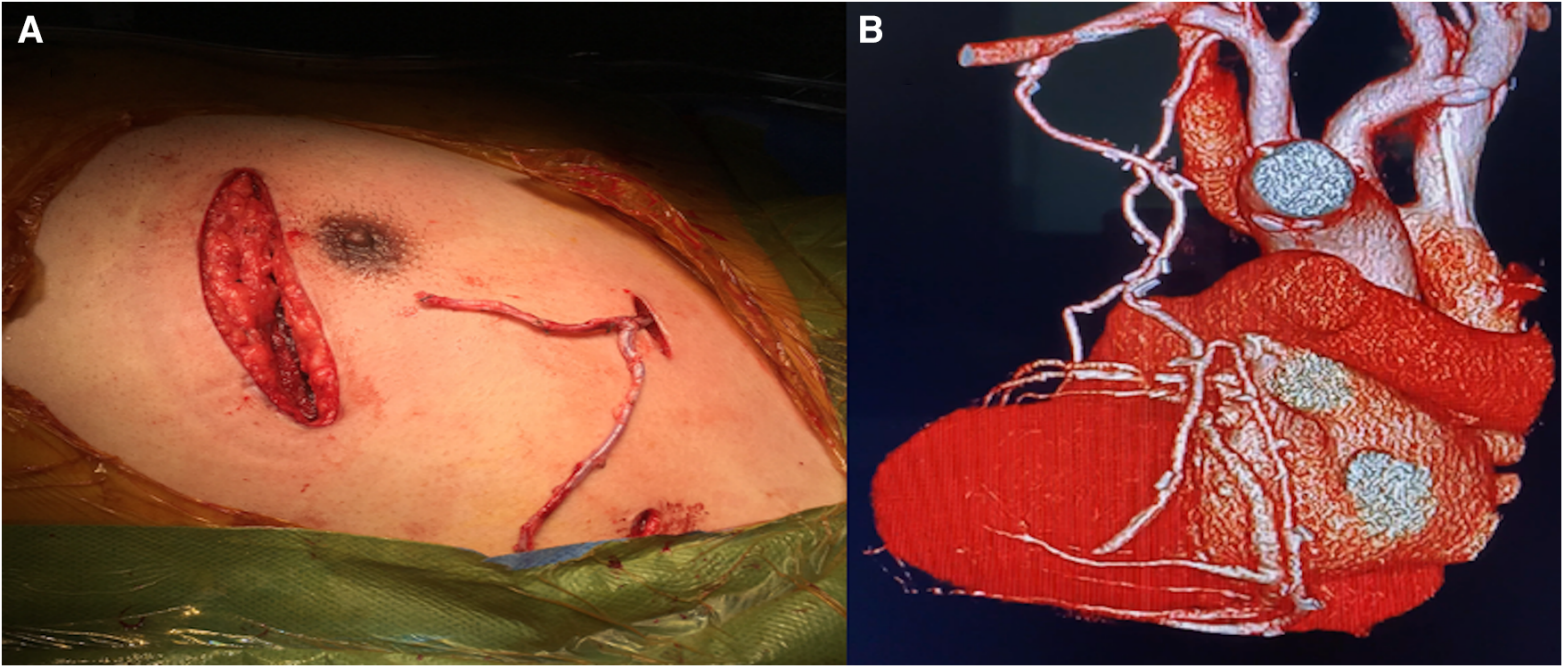
Central anastomosis made on the left subclavian artery. (**A**) Y-composite graft (saphenous vein—radial artery), and (**B**) postoperative CTA.

In MICS-OPCAB cases where harvesting RITA is challenging, LITA Y-composite grafts or the left subclavian artery (Figure 3) are good options for conduits. The Y-composite from LITA as the inflow has shown satisfactory clinical results, when the gold standard configuration of LITA-LAD is preserved (17, 18). CABG using the left axillary artery as a conduit is reported to have a 1-year patency rate of 80%–90% (19), but longer term patency rates remain uncertain. Nonetheless, we believe that the left subclavian artery is a viable option for elderly patients with severe calcification and atheroma in the ascending aorta; At our institution, we have performed >20 MICS-OPCAB with left subclavian artery grafting for such cases. In this way, MICS-OPCAB creates opportunity for more novel graft designs.

### 4.5 Proper patient selection for MICS-OPCAB

While exclusion criteria for MICS-OPCAB at our institution have already been discussed, the final decision is based on a comprehensive evaluation of the patient's condition, cardiac anatomy, and surgeon experience. Surgeons’ first few cases of MICS-OPCAB can be challenging and time consuming. To overcome this learning curve, we advise beginning with single LITA-LAD bypasses, and selecting simpler cases (such as patients with stable preoperative condition, normal heart size, and preserved LVEF) during early stages of performing multivessel MICS-OPCAB to quickly improve operation time (2, 20). With an established surgical technique, MICS-OPCAB can yield similar results to MS, even in cases with complex graft designs. Contrarily, MS may be a more suitable approach for patients with severely calcified coronary arteries that may require endarterectomy, because extensive exfoliating manipulation is not ideal for MICS. Endarterectomy was not performed in any MICS cases in our study.

### 4.6 Study limitations

Our study is a retrospective, nonrandomized analysis from a single medical center, where all MICS-OPCAB were performed by one surgeon. PSM was based on preoperational patient characteristics, with several unmeasured confounders. Assessing post-operation outcomes was limited, especially after discharge, because follow-up CTA is generally not done due to a lack of insurance reimbursement in Thailand. That's why our mid-term follow-up data primarily focused on the rates of MACCE and survival. Clinical examinations were conducted for outpatients, with coronary evaluation pursued for patients presenting with symptoms of cardiac ischemia. However, long-term follow-up data for coronary CTA of MICS-OPCAB were lacking. Further research on mid to long-term results is necessary. Furthermore, a simple comparison of patency rates couldn't be made because the median CTA follow-up dates of the MICS and MS groups were quite different and varied.

## 5 Conclusion

Provided that there is proper patient selection, MICS-OPCAB can provide good short to mid-term results similar to those of conventional OPCAB.

## Data Availability

The original contributions presented in the study are included in the article/Supplementary Material, further inquiries can be directed to the corresponding author.
